# Sensitive scalp syndrome in scalp psoriasis: Prevalence, correlates, and quality-of-life impact

**DOI:** 10.1016/j.jdin.2025.11.001

**Published:** 2025-11-10

**Authors:** Nguyen Pham, Chuyen Thi Hong Nguyen, Trung The Van

**Affiliations:** Department of Dermatology, University of Medicine and Pharmacy at Ho Chi Minh City, Ho Chi Minh City, Vietnam

**Keywords:** disease severity, pruritus, quality of life, scalp psoriasis, sensitive scalp syndrome

**Figure:**
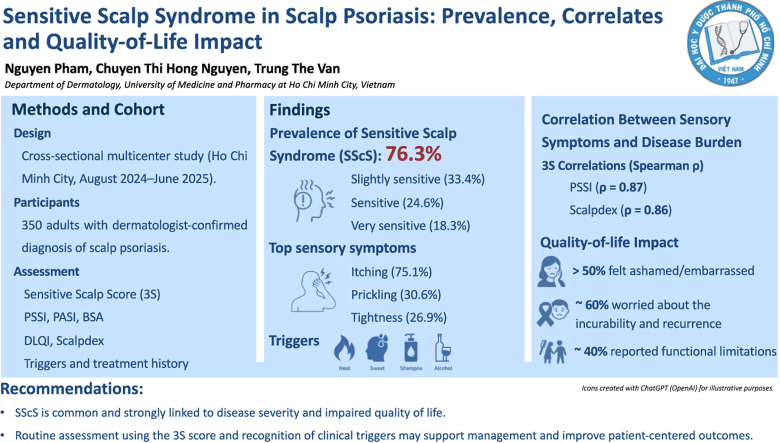

*To the Editor:* Sensitive scalp syndrome (SScS) is a common yet underrecognized feature of scalp psoriasis, which can considerably impact quality of life. SScS refers to the presence of unpleasant sensations such as itching, prickling, burning, tightness, or pain triggered by stimuli that do not typically provoke such responses.^1^ It may occur as a primary condition or secondary to scalp disorders such as psoriasis, seborrheic dermatitis, or atopic dermatitis.^2^ SScS was measured using the validated 3S scale (itching, prickling, tightness, pain, burning; scored 0-4) and categorized from nonsensitive to very sensitive.^3^^,^^4^ This is a multicenter cross-sectional study (August 2024-June 2025) in Ho Chi Minh City enrolling adults (≥18 years) with dermatologist-confirmed scalp psoriasis to investigate the prevalence, correlates, and quality of life in patients with SScS. Assessments included the Sensitive Scalp Score (3S), the psoriasis scalp severity index (PSSI), the psoriasis area and severity index, body surface area, the Dermatology Life Quality Index, and Scalpdex. Data on triggers, treatment history, and satisfaction were collected.

Among patients (median age, 49 years; interquartile range, 37-60), 70.6% were males, with a median disease duration of 10 years, and thick plaque was the most frequent morphology. Of the 350 patients, 76.3% had SScS, categorized as “slightly sensitive” (33.4%), “sensitive” (24.6%), and “very sensitive” (18.3%) (Fig 1). Itching (75.1%) was the most frequently reported symptom, followed by prickling (30.6%) and tightness (26.9%). Spearman correlation analysis demonstrated a strong positive correlation between itching and SScS (ρ = 0.92), followed by prickling (ρ = 0.72), tightness (ρ = 0.58), burning (ρ = 0.49), and pain (ρ = 0.41). (Supplementary Table I, available via Mendeley at https://data.mendeley.com/datasets/2hmmw6dk84/1) The 3S score showed a strong correlation with key severity indices, including PSSI (ρ = 0.87), DLQI (ρ = 0.69), and Scalpdex total score (ρ = 0.86). (Supplementary Table II, available via Mendeley at https://data.mendeley.com/datasets/2hmmw6dk84/1) According to Scalpdex scores, nearly half of the patients reported feeling ashamed or embarrassed, and over 50% worried about the incurability and recurrence of their condition. Functional limitations included difficulties with hairstyling, selecting clothing, and engaging in social interactions. These findings highlight the multifaceted burden of SScS, which extends far beyond physical symptoms (Supplementary Table III, available via Mendeley at https://data.mendeley.com/datasets/2hmmw6dk84/1).

**Fig 1:**
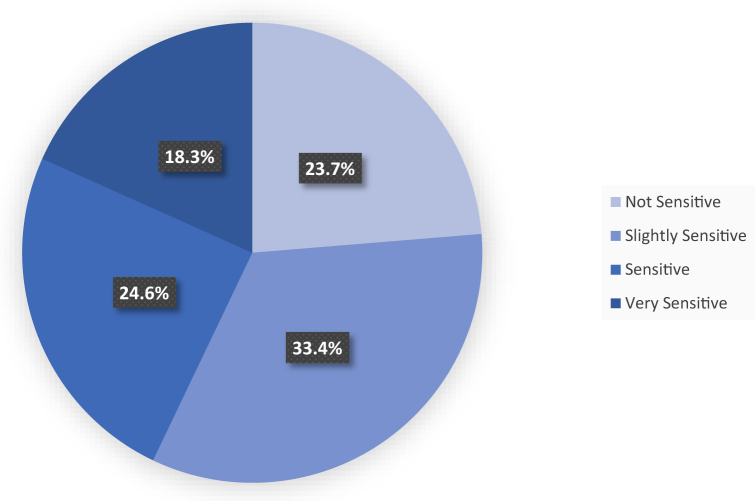
Distribution of sensitive scalp syndrome based on the 3S. The maximum total score is 20, categorizing patients into 4 groups: nonsensitive (0 points), slightly sensitive (1 point), sensitive (2-3 points), and very sensitive (greater than 3 points).

Multivariate logistic regression identified PSSI and Scalpdex as independent predictors of SScS, reinforcing the notion that sensory symptoms are intrinsically linked to disease activity and patient-perceived burden. Notably, thin desquamation morphology and facial involvement were protective, suggesting possible phenotypic or neuroanatomical variation (Table I). Several triggering factors were associated with individual symptoms of SSsS. Sweat was strongly correlated with itching (odds ratio [OR]: 2.37; *P* = .001) and burning (OR: 2.45; *P* = .043). Heat exposure was linked to itching (OR = 3.19, *P* = .010), alcohol to pain (OR = 7.68, *P* = .001), and shampoo to prickling (OR = 6.31, *P* = .030), whereas sun, stress, exercise, and spices showed no specific associations (Supplementary Table IV, available via Mendeley at https://data.mendeley.com/datasets/2hmmw6dk84/1). Limitations include the cross-sectional study design and the lack of objective neurophysiological assessments, such as transient receptor potential (TRP) channel expression or cutaneous nerve fiber density.

**Table I:** Multivariable logistic regression identifying independent predictors of sensitive scalp syndrome (*n* = 350)

Characteristics	OR (95% CI)	*P* value
Gender		
Male	1	.893
Female	1.09 (0.30-3.83)	
Age	1.00 (0.95-1.05)	.848
Age on set	0.98 (0.94-1.03)	.658
Clinical types of scalp psoriasis		
Plaque	1	**.006**
Thin desmaquation	0.23 (0.08-0.66)	
Difficult-to-treat areas		
Palms/soles	1.33 (0.31-5.67)	.700
Face	0.15 (0.02-0.86)	**.034**
Intertriginous	0.24 (0.03-1.54)	.134
Genital	3.28 (0.62-17.22)	.159
Nail	0.89 (0.36-2.18)	.811
Comorbidities		
Diabetes mellitus	1.28 (0.30-5.47)	.736
Psoriatic arthritis	1.50 (0.39-5.73)	.545
BSA	1.02 (0.98-1.07)	.250
PASI	1.01 (0.83-1.21)	.908
PSSI	1.31 (1.01-1.69)	**.036**
DLQI	0.86 (0.70-1.06)	.181
Scalpdex	1.18 (1.09-1.28)	**<.001**
Current treatment modalities		
Topical	2.46 (0.91-6.64)	.074
Conventional systemic	0.43 (0.09-2.00)	.285
Biologics	0.36 (0.10-1.25)	.110

Multivariable logistic regression was used to assess the association between clinical variables and the presence of sensitive scalp syndrome (SSCS). Values with *P* < .05 are in bold.

*OR*, Odds ratio; *BSA*, body surface area; *PASI*, psoriasis area and severity index; *PSSI*, psoriasis scalp severity index; *DLQI*, dermatology life quality index.

In conclusion, SScS is common in scalp psoriasis and strongly linked to disease severity and reduced quality of life. Routine use of tools like the 3S score and recognition of clinical triggers may optimize patient-centered care.

## Conflicts of interest

None disclosed.
